# Reversions of QuantiFERON-TB Gold Plus in tuberculosis contact investigation: A prospective multicentre cohort study

**DOI:** 10.1371/journal.pone.0285917

**Published:** 2023-08-30

**Authors:** Sandra Pérez-Recio, Maria D. Grijota-Camino, Luis Anibarro, Ramón Rabuñal-Rey, Josefina Sabria, Paloma Gijón-Vidaurreta, Virginia Pomar, Mercedes García-Gasalla, Ángel Domínguez-Castellano, Matilde Trigo, María Jesús Santos, Alba Cebollero, Sara Rodríguez, Esther Moga, Anton Penas-Truque, Carmen Martos, M. Jesús Ruiz-Serrano, Erika I. Garcia-de-Cara, Fernando Alcaide, Miguel Santin

**Affiliations:** 1 Tuberculosis Unit, Department of Infectious Diseases, Bellvitge University Hospital-Bellvitge Biomedical Research Institute (IDIBELL), L’Hospitalet de Llobregat, Barcelona, Spain; 2 Department of Fundamental and Medical-Surgical Nursing, University of Barcelona, L’Hospitalet de Llobregat, Barcelona, Spain; 3 Tuberculosis Unit, Department of Internal Medicine, Complexo Hospitalario Universitario de Pontevedra, Pontevedra, Spain; 4 Infectious Diseases Unit, Department of Internal Medicine, University Hospital Lucus Augusti, Lugo, Spain; 5 Tuberculosis Unit, Hospital Moisès Broggi, Sant Joan Despí, Barcelona, Spain; 6 Clinical Microbiology and Infectious Diseases, Hospital General Universitario Gregorio Marañon, Madrid, Spain; 7 Instituto de Investigación Sanitaria Gregorio Marañón (IiSGM), Madrid, Spain; 8 Infectious Diseases Unit, Department of Internal Medicine, Hospital de la Santa Creu i Sant Pau, Barcelona, Spain; 9 Department of Internal Medicine, Hospital Universitari Son Espases, Palma, Spain; 10 Balearic Islands Health Research Institute (IdISBa), Palma, Spain; 11 Clinic Unit of Infectious Diseases and Preventive Medicine, Hospital Virgen Macarena, Sevilla, Spain; 12 Microbiology Department, Complexo Hospitalario Universitario de Pontevedra, Pontevedra, Spain; 13 Tuberculosis Unit, Hospital Lucus Augusti, Lugo, Spain; 14 Department of Clinical Analysis, CLILAB Diagnostics Laboratory, Vilafranca del Penedés, Barcelona, Spain; 15 Department of Immunology, Hospital de la Santa Creu i Sant Pau, Biomedical Research Institute Sant Pau (IIB Sant Pau), Barcelona, Spain; 16 Centro de Investigación Biomédica en Red (CIBER) de Enfermedades Respiratorias- CIBERES (CB06/06/0058), Madrid, Spain; 17 Department of Microbiology, Bellvitge University Hospital—Bellvitge Biomedical Research Institute (IDIBELL), L’Hospitalet de Llobregat, Barcelona, Spain; 18 Department of Pathology and Experimental Therapy, University of Barcelona, L’Hospitalet de Llobregat, Barcelona, Spain; 19 Department of Clinical Sciences, University of Barcelona, L’Hospitalet de Llobregat, Barcelona, Spain; 20 Centre for Biomedical Research in Infectious Diseases Network (CIBERINFEC), Instituto de Salud Carlos III, Madrid, Spain; Showa University Fujigaoka Hospital, JAPAN

## Abstract

**Background:**

Interferon-y Release Assays (IGRA) reversions have been reported in different clinical scenarios for the diagnosis of tuberculosis (TB) infection. This study aimed to determine the rate of QuantiFERON-TB Gold Plus (QFT-Plus) reversions during contact investigation as a potential strategy to reduce the number of preventive treatments.

**Methods:**

Prospective, multicentre cohort study of immunocompetent adult contacts of patients with pulmonary TB tested with QFT-Plus. Contacts with an initial positive QFT-Plus (QFT-i) underwent a second test within 4 weeks (QFT-1), and if negative, underwent a repeat test 4 weeks later (QFT-2). Based on the QFT-2 result, we classified cases as sustained reversion if they remained negative and as temporary reversion if they turned positive.

**Results:**

We included 415 contacts, of whom 96 (23.1%) had an initial positive test (QFT-i). Following this, 10 had negative QFT-1 results and 4 (4.2%) of these persisted with a negative result in the QFT-2 (sustained reversions). All four sustained reversions occurred in contacts with IFN-γ concentrations between ≥0.35 and ≤0.99 IU•mL^-1^ in one or both QFT-i tubes.

**Conclusion:**

In this study, TB contact investigations rarely reveal QFT-Plus reversion. These results do not support retesting cases with an initial positive result to reduce the number of preventive treatments.

## Introduction

Currently, no method exists to confirm whether positive Interferon-y Release Assay (IGRA) results in tuberculosis (TB) contact tracing are due to recent exposure or more distant infection. A pragmatic approach in clinical practice is to consider all positive cases as being caused by recent exposure. This unavoidably leads to unnecessary preventive treatment in many cases. Interestingly, repeating IGRA tests leads to frequent conversions and reversions [1]. Since there is not a gold standard for latent TB infection diagnosis, the meaning of such conversions and reversions are rather speculative. In contact tracing studies, part of these conversions may be due to a de novo infection. Likewise, some of the reversions may be consequence of clearing the infection [2, 3]. However, since a significant proportion of them occur with values near to the threshold of positivity of the test–uncertainty zone- suggests that in many instances they are due to biological variability of the technique. Reversions with QuantiFERON-TB Gold (QFT) serial testing when investigating TB contacts have been reported in different studies [4–7], though the extent to which these reversions represent clearance of the infection or inconsistency of the test is unknown. If these observations could be proved consistently, repeating the initial positive QFT test may help to reduce the preventive treatments prescribed. However, most existing data come from studies conducted in populations with diverse risk and susceptibility to TB infection and not specifically designed to study reversions [4–9]. In addition, these studies were performed with the no longer used QFT In-tube (QFT-GIT) test. In here, we aimed to determine the rate of QuantiFERON^®^-TB Gold Plus (QFT-Plus) reversions to see if the number of preventive therapies prescribed in contact investigation could be reduced.

## Materials and methods

### Study design

We conducted a prospective, multicentre cohort study of TB contacts tested with the QFT-Plus at eight health centres in Spain: Bellvitge University Hospital (L’Hospitalet de Llobregat, Barcelona), Complexo Hospitalario de Pontevedra, University Hospital Lucus Augusti (Lugo), Hospital Moisès Broggi (Sant Joan Despí, Barcelona), Hospital General Universitario Gregorio Marañón (Madrid), Hospital de la Santa Creu i Sant Pau (Barcelona) Hospital Universitari Son Espases (Palma de Mallorca) and Hospital Universitario Virgen Macarena (Sevilla). Patients were recruited between March 1^st^, 2019, and June 30^th^, 2021. Eligible subjects were those contacts of patients with pulmonary or laryngeal TB, aged ≥ 18 years, who attended at TB units of every healthcare centre for evaluation as a part of a TB contact investigation. Exclusion criteria were HIV infection, debilitating chronic conditions (end-stage renal failure or liver cirrhosis stage Child-Pugh B or C), or treatment with immunosuppressive drugs, or chemotherapy in the previous 3 months. Previous diagnosis of latent or active TB was also an exclusion criteria.

At the first visit, participants underwent clinical assessment and QFT-Plus testing. All contacts with a positive QFT-Plus result also underwent chest X-ray and respiratory samples were collected if active TB was suspected. Patients who were diagnosed of active TB were excluded. Contacts with initial negative QFT-Plus were managed according to physician’s practice at each centre and no further follow-up was carried out after the result of the first test. Contacts who had an initial positive test (QFT-i) underwent a second test within the following 4 weeks (QFT-1). If the QFT-1 was positive, we considered it a persistent positive QFT-Plus result. If the QFT-1 was negative, it was repeated 4 weeks later (QFT-2): if the QFT-2 was negative it was considered a sustained reversion and if it was positive it was considered a temporary reversion. Participants with persistent positive QFT-Plus results and those with temporary reversions where considered as having TB infection and required preventive therapy. Participants with sustained reversion were considered as no having TB infection and decision on treatment of these contacts was left to the discretion of the treating physician. No participant started preventive therapy until the last test was performed. All participants were followed-up until the last QFT was performed or until the preventive therapy was finished if it was indicated.

Concerning to contacts, we collected data on demographics, bacillus Calmette-Guérin (BCG) vaccination status, extension and date of initiation and end of the contact period, and QFT-Plus qualitative and quantitative results. Contacts were categorized in three groups based on the likelihood of transmission. High transmission risk (close contact) was considered when the exposure occurred in an favourable environment to transmission, for at least 6 hours per day, and included household contacts, members of the same classroom or premises, close work contacts, intimate friends, and contacts in nursing homes or prisons. Medium risk (frequent contact) was considered when exposure occurred in an environment that favoured transmission for less than 6 hours a day, without the conditions for high risk. Finally, low transmission risk (sporadic contact) was reserved for non-daily contacts [10]. Concerning to the index case, we collected data on the type of TB (cavitary or non-cavitary and bacilliferours or non-bacilliferous TB) and the diagnostic technique used (culture and/or nucleic acid amplification test [NAAT]). De-identified anonymous clinical data were collected prospectively and handled using REDCap (Research Electronic Data Capture) tools [11].

Samples were processed according to local standard clinical practice. The research protocol did not call for sample storage. QuantiFERON^®^-TB Gold Plus was performed in accordance with the manufacturer’s instructions. Four centres changed from the QFT-Plus ELISA [12] to the LIAISON QFT-Plus [13] over the study period. None individual participants included in the analysis were tested by different QFT-Plus techniques. We used the 0.35–0.99 IU·mL^-1^ positive borderline range of the QFT-Plus in line with other low-incidence TB European settings [14].

### Statistical analysis

The sample size was determined based on the assumption that around 35% of contacts would have a positive initial result [15]. Assuming a prevalence of 35% negative results for the first repeated test (QFT-1) [1, 4], with 80% of these remaining negative in QFT-2, 355 contacts were needed to obtain a sample of 113 with a positive initial result (QFT-i). This would allow the estimation of 25% sustained reversion with an 8% precision and a 95% confidence interval (CI) of 17%–33%, allowing for 10% expected losses.

Categorical variables are presented as the number of cases and percentages, and continuous variables are presented as the mean and standard deviation or median and interquartile range (IQR) according to the distribution. Continuous variables were compared using Student t test or Mann-Whitney U test where appropriate. Fisher exact test or Pearson chi-square test was applied to assess the relationship between categorical variables. Statistical analysis was performed with the statistical package R, version 3.4.1.

### Ethics statement

This prospective study was reviewed and approved by the ethics committee of Bellvitge University Hospital (approval number PR165/18) and conducted in accordance with the Declaration of Helsinki. Written informed consent was obtained from all participants.

## Results

**Fig 1:**
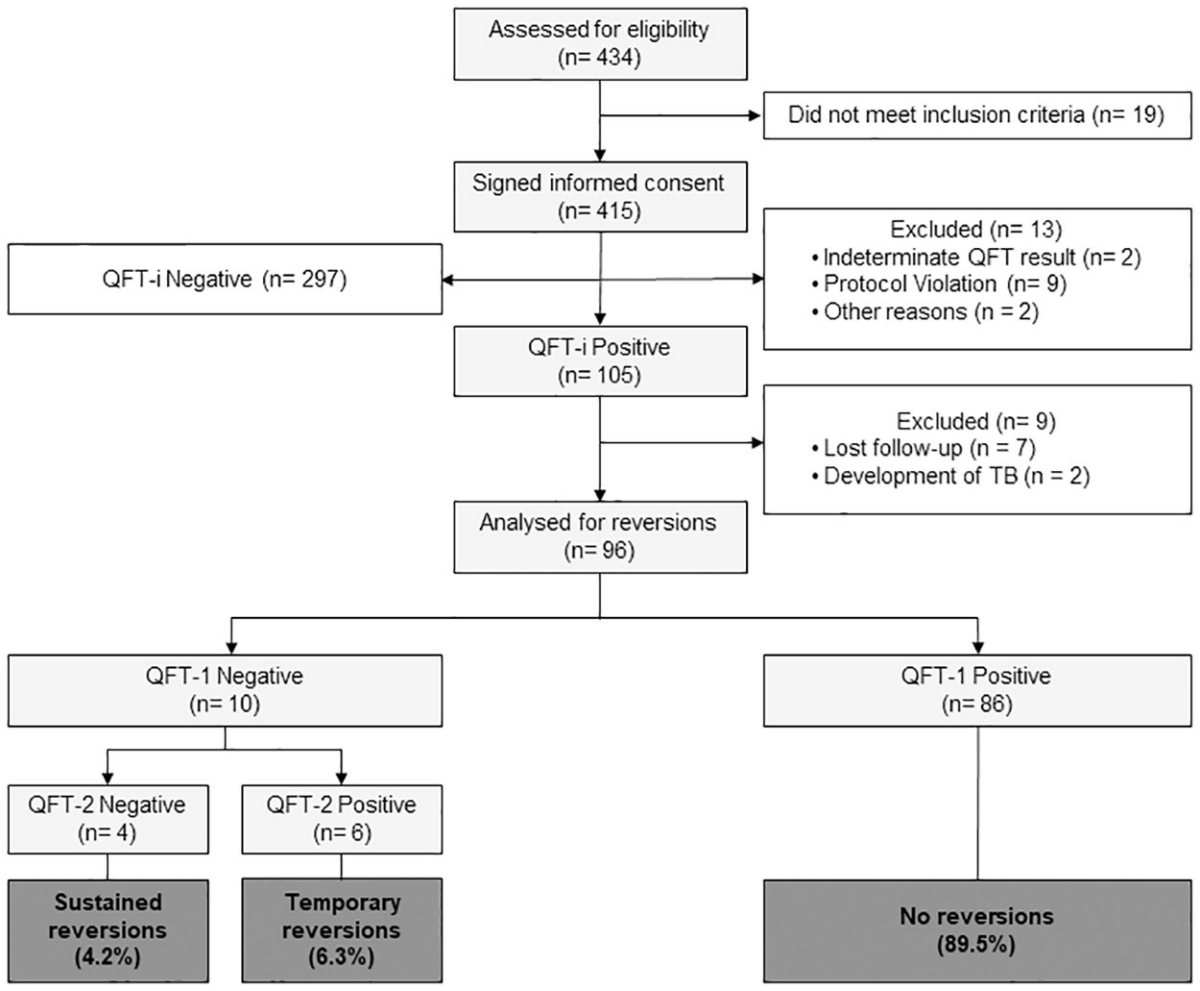
Flowchart of the study. Selection of participants and main results. Abbreviations: QFT-i, initial QuantiFERON Gold Plus; QFT-1, week 4 QuantiFERON Gold Plus; QFT-2, week 8 QuantiFERON Gold Plus.

As the 26.1% prevalence of positive QFT-Plus results was lower than the 35% expected, we finally recruited 415 contacts. Of these, 297 had a negative QFT-i and therefore they were removed from subsequent analyses and 13 were excluded for different reasons. Of the 105 individuals with a positive QFT-i result, 9 were excluded, leaving 96 for the reversion analysis. See Fig 1 for patient flow diagram. The main characteristics of the 96 contacts with positive initial QFT-Plus result are showed in the Table 1.

**Table 1:** Main characteristics of the 96 contacts with a positive initial QFT-Plus result.

	Non-reversion group n = 86	Reversion group n = 10
**Age, median [IQR 25%;75%]**	48 [39;56]	57.5 [52.25;68.5]
**Gender, N (%)**		
Male	40 (46.5)	3 (30.0)
Female	46 (53.5)	7 (70.0)
**TB incidence in country of birth, N (%)**		
≥25 × 10^5^	31 (36.0)	1 (10.0)
<25 × 10^5^	55 (64.0)	9 (90.0)
**BCG vaccination status, N (%)**		
Yes	33 (38.4)	4 (40.0)
No	46 (53.5)	4 (40.0)
Unknown	7 (8.1)	2 (20.0)
**Type of contact, N (%)**		
Close	43 (50.0)	4 (40.0)
Frequent	23 (26.7)	3 (30.0)
Sporadic	19 (22.1)	3 (30.0)
Unknown	1 (1.2)	0
**Days of exposure, median [Q1;Q3]**	91.5 [61.0;123]	63.0 [15.0;91.8]
**Chest X-ray of the index case**		
Cavitary TB	51 (59.3)	5 (50.0)
Non-cavitary TB	35 (40.7)	5 (50.0)
**Diagnosis of TB of the index case**		
By culture	25 (29.1)	6 (60.0)
By NAAT	8 (9.3)	1 (10.0)
By both techniques	53 (61.6)	3 (30.0)
**AFB smear-positive sputum**	55 (64.0)	4 (40.0)

Abbreviations: TB, tuberculosis, BCG, Bacillus Calmette–Guérin; AFB, acid-fast bacilli; MTC, Mycobacterium tuberculosis complex; NAAT, nucleic acid amplification test; QFT-i, initial QuantiFERON Gold Plus; TB, Tuberculosis.

In the QFT-1, 86 (89.6%) contacts tested positive again and were considered persistent positive results, while only 10 contacts (10.4%) tested negative. Twenty-four of the 96 (25%) contacts had a QFT-i result within the borderline range of whom 10 (41.7%) reverted (4 sustained reversion, and 6 had temporary reversion), and 14 (58.3%) did not revert. Table 2 shows the quantitative results of all QFT-Plus tests for the 10 contacts with a negative QFT-1 result, which reveals 4 cases of sustained reversion and the 6 cases of temporary reversion. The median TB1–Nil and TB2–Nil IFN-γ concentrations in the QFT-i for the 10 individuals with any reversion (sustained or temporary) were 0.5 (interquartile range [IQR] 0.44;0.55) and 0.53 (IQR 0.37;0.66) IU·mL^-1^, respectively; the corresponding values for those without reversion were 3.78 (IQR 1.55;5.66) and 3.75 (IQR 1.7;5.54) IU·mL^-1^ (p < 0.001 for the median TB1–Nil and p < 0.001 for the median TB2–Nil IFN-γ concentrations). Note that only one reverter (10%) had a QFT-i result >0.99, the upper limit of the positive borderline range, and only one of the temporary reverters had a QFT-2 result >0.99, and only in TB1 tube.

**Table 2:** Interferon-γ production in QuantiFERON-TB Gold Plus tubes in 10 contacts with reversion.

Case	Sex/age	Contact	QFT-i (IU·ml^-1^)		QFT-1 (IU·ml^-1^)		QFT-2 (IU·ml^-1^)		Reversion
			TB-1	TB-2	TB-1	TB-2	TB-1	TB-2	
1	Female/70 y.	Frequent	**0.40**	0.24	0.19	0.20	0.12	0.09	Sustained
2	Female/57 y.	Sporadic	**0.77**	**0.72**	0.10	0.20	0.07	0.04	Sustained
3	Female/86 y.	Close	**0.51**	0.17	0.00	0.00	0.01	0.01	Sustained
4	Female/48 y.	Close	**0.48**	**0.59**	0.13	0.13	0.11	0.09	Sustained
5	Male/64 y.	Frequent	**0.50**	**0.62**	0.24	0.24	0.17	**0.40**	Temporary
6	Female/75 y.	Close	0.24	**0.35**	0.12	0.11	**0.46**	0.30	Temporary
7	Female/52 y.	Frequent	**0.57**	**0.67**	0.26	0.18	**0.46**	**0.41**	Temporary
8	Male/30 y.	Sporadic	**0.43**	**0.44**	0.31	0.26	**1.12**	**0.94**	Temporary
9	Female/58 y.	Close	**0.51**	**0.48**	0.20	0.17	0.31	**0.43**	Temporary
10	Male/53 y.	Sporadic	**0.99**	**1.05**	0.23	0.20	**0.35**	**0.50**	Temporary

Abbreviations: QFT-i, initial QuantiFERON Gold Plus; QFT-1, week 4 QuantiFERON Gold Plus; QFT-2, week 8 QuantiFERON Gold Plus; TB-1 & TB-2, QuantiFERON-TB Gold Plus tubs with large-length and large- plus short-length ESAT-6 and CP-10 Mycobacterium tuberculosis antigens, respectively.

Note: Bold numbers denote interferon-γ values within the borderline positive range (defined as 0.35–0.99 IU·ml-1), and bold underlined numbers denote interferon-γ values outside this range.

## Discussion

In this study, we prospectively assessed QFT-Plus reversions in a cohort of TB contacts in a low incidence TB setting. The 4.2% reversion rate was very low compared with studies published to date. In a retrospective study in Sweden [4], which included TB contacts among others, repeating positive QFT-GIT tests (initial IFN-γ concentration, 0.35–0.99 IU·mL^-1^) after 3–12 weeks yielded a reversion rate of 54.7%, and reached 59.4% in a subgroup when it was retested within 4 weeks. In a study of household contacts in India, repeating the QFT-GIT after 12 months revealed an overall reversion rate of 6.7%, which reached 20% among those with initial IFN-γ values of 0.35–0.7 IU·mL^-1^ [5]. Finally, a study of adult household contacts in South Africa reported a reversion rate of 14.5% when repeating the QFT-GIT after 6 months [6].

Only limited data exists about QFT-Plus reversion, and no data have been reported for TB contacts. A retrospective analysis of 2.2 million QFT-Plus tests found reversion rates of 24% and 30% depending on whether the test had been repeated within or after the first 30 days, respectively [16]. When comparing reversion rates among three IGRA tests repeated 3 and 6 months apart among registered village doctors in China, persistent reversion occurred in 13% for QFT-Plus, 5.9% for the QFT-GIT, and 6.5% for the T-SPOT.TB [17].

Most reversions could be explained by the biological variability of tests resulting in a cross pass from above the borderline limit. Repeating the test only once may lead to reversions, which might become positive again in a second repetition. Therefore, we tried to identify sustained reversions with negative results in two determinations at least 4 weeks apart. Whatever the reason for sustained reversion, the low rates observed do not support the systematic repetition of QFT-Plus testing for contacts with an initial positive test. Nonetheless, since 90% of reversions occurred in participants with a positive QFT-i of 0.35–0.99 IU·mL^-1^, it may be helpful to eventually retest certain contacts in case of results within this range and an unclear risk of exposure.

We do not have a convincing explanation for the discrepancy in reversion rate we found compared with studies published to date. That said, our study was conducted to confirm/refute these previous findings, which derived from studies not designed primarily for this purpose. Our study was prospectively designed with the aim of providing a definitive answer on the relevant issue of the diagnosis, and consequently need for treatment, in TB contact tracing. The other two factors explaining such discrepancy may be the more conservative definition of reversals ("two consecutive negative tests 4 weeks apart"), and the use of the QFT-Plus version, from which a different behavior from the previous QFT-TB Gold In-Tube cannot be ruled out.

In conclusion, QFT-Plus reversion occurs rarely in TB contact investigations. Therefore, we cannot advocate repeating the test in cases with an initial positive result as a potential strategy for reducing the number of preventive treatments.

## Supporting information

S1 Checklist STROBE statement—Checklist of items that should be included in reports of observational studies. (DOCX)

S1 Dataset (XLSX)
